# *In vivo* Hippocampal Serotonin Dynamics in Male and Female Mice: Determining Effects of Acute Escitalopram Using Fast Scan Cyclic Voltammetry

**DOI:** 10.3389/fnins.2019.00362

**Published:** 2019-04-23

**Authors:** Rachel A. Saylor, Melinda Hersey, Alyssa West, Anna Marie Buchanan, Shane N. Berger, H. Frederik Nijhout, Michael C. Reed, Janet Best, Parastoo Hashemi

**Affiliations:** ^1^Department of Chemistry and Biochemistry, University of South Carolina, Columbia, SC, United States; ^2^Department of Pharmacology, Physiology, and Neuroscience, University of South Carolina School of Medicine, Columbia, SC, United States; ^3^Department of Biology, Duke University, Durham, NC, United States; ^4^Department of Mathematics, Duke University, Durham, NC, United States; ^5^Department of Mathematics, The Ohio State University, Columbus, OH, United States

**Keywords:** serotonin, hippocampus, SSRI, FSCV, FSCAV, sex, depression

## Abstract

Depression is a highly prevalent psychiatric disorder, impacting females at a rate roughly twice that of males. This disparity has become the focus of many studies which are working to determine if there are environmental or biological underpinnings to depression pathology. The biology of depression is not well understood, but experts agree that a key neurotransmitter of interest is serotonin. Most research on basic serotonin neurochemistry, by us and others, has predominantly focused on male models. Thus, it is now critical to include female models to decipher possible fundamental differences between the sexes that may underlie this disorder. In this paper, we seek to determine any such differences using fast-scan cyclic voltammetry (FSCV) and fast-scan controlled adsorption voltammetry. These techniques allow us to probe the serotonergic system *via* measurement of evoked and ambient serotonin at carbon fiber microelectrodes (CFMs). Our data reveal no statistical differences, in the hippocampus, in female serotonin chemistry during the different stages of the estrous cycle compared to the mean female response. Furthermore, no difference was observed in evoked serotonin release and reuptake, nor ambient extracellular serotonin levels between male and female mice. We applied a previously developed mathematical model that fits our serotonin signals as a function of several synaptic processes that control the extracellular levels of this transmitter. We used the model to study potential system differences between males and females. One hypothesis brought fourth, that female mice exhibit tighter autoreceptor control of serotonin, is validated *via* literature and methiothepin challenge. We postulate that this tight regulation may act as a control mechanism against changes in the serotonin signal mediated by estrogen spikes. Importantly, this safety mechanism has no consequence for acutely administered escitalopram’s (ESCIT’s) ability to increase extracellular serotonin between the sexes. This work demonstrates little fundamental differences in *in vivo* hippocampal serotonin between the sexes, bar control mechanisms in female mice that can be observed under extraneous circumstances. We thus highlight the importance of considering sex as a biological factor in determining pharmacodynamics for personalized medical treatments that involve targeting serotonin receptors.

## Introduction

Clinical depression is more than twice as prevalent in adult females than males (Weissman and Klerman, 1977; Kessler et al., 1993; Hankin et al., 1998; Cyranowski et al., 2000; Piccinelli and Wilkinson, 2000; Grigoriadis and Robinson, 2007) and antidepressants exert varying degrees of efficacy by sex (Frackiewicz et al., 2000; Sramek and Cutler, 2011). Interestingly, most studies show this sex-dependent increased risk of depression only emerges post-puberty (Anderson et al., 1987; McGee et al., 1992; Nolen-Hoeksema and Girgus, 1994). This disparity in adult depression rates has been explained *via* environmentally induced (i.e., societal stress) or innate (fundamental biology) phenomena (Sullivan et al., 2000; Eley et al., 2004). The idea that there are inherent biological underpinnings to depression is well-debated and hypotheses that have been brought forth over the years have not had unanimous acceptance (Asberg et al., 1976a,b; Owens and Nemeroff, 1994). Historically, preclinical and basic research has not encompassed both sexes equally, with a strong bias toward male models. This approach was formed under the preconception that the estrous cycle confounds experimental data, however, as stated in NOT-OD-15-102 in 2015, “An overreliance on male animals and cells may obscure understanding of key sex influences on health processes and outcomes” (National Institutes of Health, 2015). Henceforth, there is a strong emphasis on including male and female mice in all basic and preclinical studies.

We are in a good position to study intrinsic neurochemical differences that may drive depression and antidepressant actions in male and female animal models since we can monitor sub-second changes in serotonin in real time, *in vivo*. Serotonin is a well-established transmitter of interest to depression and antidepressant activity in both sexes (Piccinelli and Wilkinson, 2000; Sramek and Cutler, 2011). We monitor serotonin dynamics *in vivo* using voltammetric techniques, fast-scan cyclic voltammetry (FSCV) and fast-scan controlled adsorption voltammetry (FSCAV) at carbon fiber microelectrodes (CFMs). FSCV enables the *in vivo* monitoring of the release and reuptake of serotonin on a sub-second timescale (Hashemi et al., 2009; Wood and Hashemi, 2013; Wood et al., 2014) and FSCAV quantifies ambient serotonin concentrations on the order of tens of seconds (Abdalla et al., 2017).

In this article, we study the *in vivo* serotonin chemistry in the CA2 region of the hippocampus of male and female mice. We chose to start this study with the hippocampus because of this brain region’s heavy association with depression and antidepressant actions. For example, decreased hippocampal volume is found in human and animal models of depression (Magarinos and McEwen, 1995; Sheline et al., 1996; Bremner et al., 2000; Videbech and Ravnkilde, 2004). Furthermore, selective serotonin reuptake inhibitors (SSRIs) are shown to change hippocampal architecture *via* neurogenesis (Czeh et al., 2001; Malberg and Duman, 2003; Jayatissa et al., 2006). In our cohort of female mice, statistical differences are not found in serotonin signals between the overall female mean and the different stages of the estrous cycle. Importantly, evoked serotonin release, reuptake, and ambient serotonin in 23 female (all cycle stages) and 23 male mice are not statistically different. Potential functional differences are investigated by mathematically modeling the averaged male and female responses as a measure of several synaptic processes that regulate extracellular serotonin. Specifically, the evoked signal in male mice is postulated to have higher input and the signal in female mice is hypothesized to undergo tighter autoreceptor control. We provide validation of the model’s autoreceptor hypothesis in female mice *via* administration of methiothepin, a non-selective serotonin receptor antagonist with high affinity for the serotonin autoreceptors. We put forth that this stronger autoreceptor control may act as a safety mechanism against serotonin-mediating estrogen spikes. To understand whether these autoreceptor effects are consequential for SSRI response, we compare administration of acute doses of the SSRI, escitalopram (ESCIT) to male and female cohorts of mice, some of which are pretreated with methiothepin. The percent change in serotonin reuptake is less dramatic across all doses of ESCIT in females but this effect is independent of autoreceptor antagonism.

In summary, serotonin chemistry, in the hippocampus, during the different stages of the estrous cycle is not different from the mean in female mice and the control evoked release, reuptake, and ambient serotonin are not statistically different between the sexes. On the microanalysis level, differences in serotonin regulation between male and female mice may lie, in part, in autoreceptor regulation. This finding is especially useful when considering pharmacodynamics for personalized medical treatments that involve serotonin receptors.

## Materials and Methods

### Chemicals and Reagents

Calibration solutions were prepared by dissolving serotonin hydrochloride (Sigma–Aldrich Co., St. Louis, MO, United States) in Tris buffer to produce solution concentration of 10, 25, 50, and 100 nM. Tris buffer consisted of: 15 mM H_2_NC(CH_2_OH)_2_ HCl, 140 mM NaCl, 3.25 mM KCl, 1.2 mM CaCl_2_, 1.25 mM NaH_2_PO_4_.H_2_O, 1.2 mM MgCl_2_, and 2.0 mM Na_2_SO_4_ (Sigma–Aldrich Co., St. Louis, MO, United States) in deionized water and pH adjusted to 7.4. ESCIT oxalate (≥98, HPLC) (3, 10, or 30 mg kg^-1^) from Sigma–Aldrich (St. Louis, MO, United States) and methiothepin mesylate salt (≥98, HPLC) also from Sigma–Aldrich (St. Louis, MO, United States) were individually dissolved in sterile saline (Hospira, Lake Forest, IL, United States) and administered *via* intraperitoneal (i.p.) injection at a volume of 5.0 ml kg^-1^ of animal weight. Liquion (LQ-1105, 5% by weight Nafion^TM^) was purchased from Ion Power Solutions (New Castle, DE, United States).

### Electrode Fabrication

Voltammetric analysis of serotonin was performed as described previously (Hashemi et al., 2009; Wood and Hashemi, 2013; Wood et al., 2014). Briefly, CFMs were constructed by aspirating 7 μm carbon fibers (Goodfellow Corporation, Coraopolis, PA, United States) into glass capillaries (0.4 mm internal diameter, 0.6 mm outer diameter, AM Systems, Carlsborg, WA, United States). A vertical pipette puller (Narishige Group, Tokyo, Japan) was employed to create a carbon-glass seal. Subsequently, the exposed carbon fiber was cut to 150 μm and silver paint was used to forge an electrical connection to a connection pin. Finally, electrodes were electrodeposited with Nafion^TM^ as described previously (Hashemi et al., 2009).

### Animal and Surgical Procedures

All animal procedures and protocols were performed in accordance with regulations of the Institutional Animal Care and Use Committee (IACUC) at the University of South Carolina, which operates with accreditation from the Association for Assessment and Accreditation of Laboratory Animal Care (AAALAC). Male and female C57BL/6J mice (Jackson Laboratory, Bar Harbor, ME, United States), 6–12 weeks old and weighing 18–25 g, were group housed, had constant access to food and water, and were kept on a 12 h light/dark cycle (lights off at 7:00 and on at 19:00). We chose to include mice from this broad age range since we found no statistical differences in mice aged 6–8 and 9–12 weeks (Supplementary Figure S1). Female mice were selected at random, without regard to their estrous cycle. In the estrous cycle experiments, vaginal smears were collected after the conclusion of the experiment and cycle determine according to Caligioni (Supplementary Figure S2) (Caligioni, 2009). Estrous cycle determination was limited to a single-day cell analysis at the end of the neurochemical analysis in order to limit stress to the animal that would likely alter FSCV/FSCAV serotonergic responses. To induce and maintain anesthesia, 25% w/v urethane [Sigma–Aldrich Co., dissolved in 0.9% NaCl solution (Hospira)] was injected i.p. (7 μl/g of body weight). Mouse body temperature was maintained using a heating pad (Braintree Scientific, Braintree, MA, United States). Stereotaxic surgery (David Kopf Instruments, Tujunga, CA, United States) was performed, and all coordinates were taken in reference to bregma. A Nafion^TM^-modified CFM was lowered into the CA2 region of the hippocampus (AP: -2.91, ML: +3.35, DV: -2.5 to -3.0) (Franklin, 2013) or into the medial prefrontal cortex (mPFC) (AP: +1.7, ML: -0.2, DV: -2.2 to -2.9) (Franklin, 2013)and adjusted in the dorsal/ventral plane until a serotonin signal was observed. A stimulating electrode (insulated stainless steel, diameter: 0.2 mm, untwisted, Plastics One, Roanoke, VA, United States) was placed into the medial forebrain bundle (AP: -1.58, ML: +1.00, DV: -4.8) (Franklin, 2013) and a pseudo Ag/AgCl reference electrode, created by electroplating chloride (30 s in 0.1 M HCl at 5 V) onto a silver wire, was placed into the contralateral hemisphere.

### Data Collection

FSCV and FSCAV were performed using a Dagan potentiostat (Dagan Corporation, Minneapolis, NM, United States), WCCV 3.06 software (Knowmad Technologies LLC, Tucson, AZ, United States) and either a Dagan or Pine Research headstage (Pine Research Instrumentation, Durham, NC, United States). For FSCV collection, the “Jackson” serotonin waveform (Jackson et al., 1995) was applied to the electrode at a scan rate of 1000 V s^-1^ and at a frequency of 10 Hz. To evoke serotonin release, a biphasic stimulation was applied through a linear constant current stimulus isolator (NL800A Neurolog, Medical Systems Corp, Great Neck, NY, United States) with the following parameters: 60 Hz, 360 μA each, 2 ms in width, and 2 s in length. Upon completion of data collection, a high voltage was applied to the working electrode to lesion the tissue surrounding the electrode for electrode placement verification using histology.

For basal experiments, control evoked files were collected followed by the methodology being switched to FSCAV. For FSCAV collection, the serotonin waveform was applied at 100 Hz for 2 s, followed by a period of controlled adsorption where the potential was held at 0.2 V for 10 s, lastly the serotonin waveform was reapplied at 100 Hz, as described in Abdalla et al. (2017). Thirty files (at one file per minute) were collected as control files. Following control files, an i.p. injection of saline was administered and 30 more files of FSCAV were collected. Animals were then administered ESCIT (10 mg kg^-1^) i.p. and 60 files post-ESCIT were collected. The system was then switched back to traditional FSCV and four post-basal stimulation files were collected. Electrodes were then removed and underwent a post calibration in which 10 files were collected with the electrode in solutions of 10, 25, 50, and 100 nM solutions of serotonin. A dose response was also conducted using FSCV, as previously described, in which male and female mice were administered either 3, 10, or 30 mg kg^-1^ ESCIT and four control files averaged together were then compared with stimulated release 30 min post-ESCIT.

### Data Analysis and Statistics

Digital filtering (zero phase, Butterworth, 5 kHz low-pass) was accomplished within the WCCV software. For FSCV analysis, signals were smoothed using WCCV software, the cyclic voltammogram (CV) taken for serotonin identification, and the current vs. time (IT) trace extracted to visualize the release and reuptake of serotonin. Four evoked events, with 10 min between each event, were averaged for each individual mouse to establish a control evoked signal. A previously established calibration factor (49.5 ± 10.2 nA/μM) was used to convert current into concentration. For FSCAV analysis, the third CV after the reapplication of the waveform was selected for quantification, and the peak occurring approximately between 0.4 and 0.85 V was integrated to determine the charge value (pC). Post calibrations of each electrode, plotting charge (pC) vs. [serotonin] (nM), were used to determine basal concentration.

For FSCV data, four IT curves were averaged for each animal to establish a control. The average for each individual animal was then combined with the other animals in the group to determine an overall group average. The standard error of the mean (SEM) was calculated using the average IT for each animal (*n* = # animals). To determine significance between two points, a two-tailed student’s *t*-test was utilized (*p* < 0.05).

To determine the *t*_1/2_, a code was custom written in excel to fit the reuptake component of the curve and calculate the time taken to reach half of the maximum amplitude. The number of files with a concentration of less than zero was used to quantify the “dip” below baseline, associated with autoreceptors, which will be covered in more detail below. Determination of the percent reuptake change following ESCIT is explained in Section “Modeling.”

Data were excluded based on the criteria outlined herein. For all experiments, the evoked signal CV was compared to well-established *in vivo* and *in vitro* serotonin CVs and signals in which the CVs did not contain the characteristic serotonin redox peaks were excluded. Animals which did not survive the full experiment or whose drug injection did not elicit a response were removed. Signals which did not return to baseline or were otherwise unstable, as well as those which were outside the normal range, as determined by a Q-test, were removed. Data which contained a peak resulting from the stimulation electrode touching the skull that masked, delayed, or minimized the serotonin response (stimulation glitch) were excluded. According to these criteria, 10 animals were excluded, which accounts for 8% of the total number of animals used. All other data were included and all raw evoked data are shown in Figure 2.

To determine the number of animals required for observing significant differences in serotonin signals, we employed a power analysis (Charan and Kantharia, 2013). The following formula was used to calculate a quantitative endpoint for the sample size required to compare two groups.

$$\mathrm{Sample}\;\mathrm{size}=2\;{\mathrm{SD}}^{2}\;{\left(Z^{\alpha /2}+Z^{\beta }\right)}^{2}/d^{2}$$

The pooled standard deviation from the sample data was 0.83 and Cohen’s d was calculated as 1.76. The Z^α/2^ term was 1.96 (from Z table) as a type 1 error of 5% and the Z^β^ was 0.842 (from Z table) at 80% power. This power analysis resulted in a *n* = 3.5. The sample size corrected for exclusion was calculated using *n* = 3.5 and a percent loss of animals as 8%, showing that about 3.8 animals were required.

### Modeling

A previously presented mathematical model was used to model the average male and female evoked responses:

$$\frac{d[S(t)]}{dt}=R(t)(1-A(t))-\alpha \frac{V_{\max1}[S(t)]}{k_{m1}+[S(t)]}-\beta \frac{V_{\max2}[S(t)]}{k_{m2}+[S(t)]}$$

*S*(*t*) is the concentration of serotonin in the extracellular space, *R*(*t*) is the release rate of the serotonin neurons in the hippocampus near the electrode that rises briefly after stimulation and then returns to baseline, and *A*(*t*) represents the strength of the autoreceptor effect caused by rising serotonin in the extracellular space [the higher *A*(*t*) the more serotonin release is inhibited] (Wood et al., 2014). The first negative term represents reuptake resulting from Uptake 1 transporters, the serotonin transporters (SERTs), with *V*_max1_ = 19.25 nM s^-1^ and *K*_m1_ = 5 nM. The second term represents reuptake *via* Uptake 2 transporters [dopamine transporters (DATs), norepinephrine transporters (NETs), and organic cation transporters (OCTs)] with *V*_max2_ = 780 nM s^-1^ and *K*_m2_ = 170 nM. We modeled and discussed Uptake 1 and Uptake 2 in detail previously (Wood et al., 2014). Briefly, Uptake 1 is high affinity but low efficiency serotonin transport (Shaskan and Snyder, 1970) while Uptake 2 is low affinity, high efficiency serotonin transport (Daws et al., 2013; Horton et al., 2013). Thus, at serotonin concentrations well above the basal level, Uptake 2 is primarily responsible for serotonin removal from the extracellular space, but low concentrations, closer to the steady state, Uptake 2 has little effect on reuptake of serotonin. For the purpose of our simulations, we assume the basal steady state is 60 nM, roughly the mean of measured basal levels (see below), and the parameter beta decreases from 0.05 above 82 nM linearly to zero at 62 nM, reflecting the properties of Uptake 2. In some simulations, the concentration cutoffs 82 and 62 nM are slightly varied to fit the experimental data. In all our simulations, α = 1.

We believe that stimulation of the MFB causes antidromic spikes that stimulate the dorsal raphe nucleus (DRN). The increased firing of DRN neurons increases the release rate, *R*(*t*), in the hippocampus. Before stimulation, we assume that *R*(*t*) is a constant, *R*_0_, chosen so that the basal steady state is 60 nM. After stimulation, *R*(*t*) rises linearly for 1 s followed by decay back to *R*_0_ linearly over 2 s. This value can be varied to reflect the different release rates produced by slight differences in the stimulation of the MFB. The parameter *r* scales how high above *R*_0_ the release rate goes, with *r* = 1 indicating an increase of release rate of 40 nM s^-1^. We previously showed that the autoreceptor effects are long-lasting (up to 30 s) and continue after both *R*(*t*) and *S*(*t*) have returned to baseline (Wood et al., 2014). This longer lasting autoreceptor effect drives the serotonin concentration below baseline after most stimulations.

The model was further used to calculate estimates of the percentage decrease of *V*_max2_ caused by different doses of ESCIT in male mice and female mice (Figure 4). These calculations were carried out assuming that most of the initial decrease of serotonin in the extracellular space is caused by the Uptake 2 transporters. This assumption is supported by our previous work (Wood et al., 2014). The experimental data give us the evoked serotonin response before ESCIT (control curve) and the evoked serotonin response 30 min following the administration of ESCIT (dose curve). As the *K*_m2_ of Uptake 2 is known, the control signal was used to calculate the value of the effective *V*_max2_ for the control signal. The process was repeated for the ESCIT signal to calculate the effective *V*_max2_ for the ESCIT signal. Figure 4 reports the percentage change from our estimate of *V*_max2_ for control to our estimate of *V*_max2_ 30 min following the administration of ESCIT.

## Results

### Serotonin During the Different Stages of the Estrous Cycle

**FIGURE 1:**
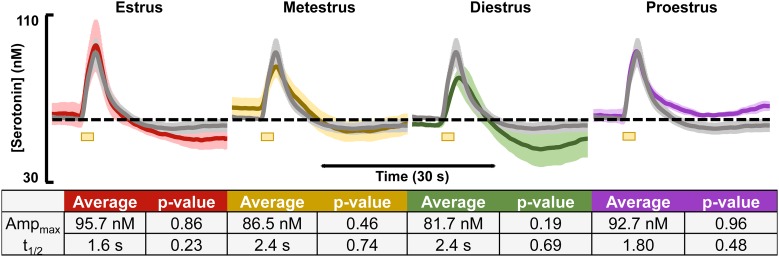
Serotonin signals, incorporating respective basal levels, from each of the four estrous cycles in female mice are shown. The average from female mice in the estrus (red, *n* = 4), metestrus (yellow, *n* = 4), diestrus (green, *n* = 3), and proestrus (purple, *n* = 3) stages are shown. A table showing the average amplitude and *t*_1/2_ for each stage of the female mouse cycle along with the *p*-value resulting from a two-tailed *t*-test is seen below. Significance was defined as *p* < 0.05.

Evoked and basal serotonin was measured during each stage of the estrous cycle in the CA2 region of the hippocampus in female mice (images verifying stage of cycle are in Supplementary Figure S2). The basal serotonin concentrations were added to the respective animals’ evoked response and are displayed in the colored traces in Figure 1 for each stage of the estrous cycle (*n* = 3–4). The gray traces in Figure 1 are the averaged female responses (blinded for stage of estrous cycle, *n* = 10). When comparing the data from each stage of the cycle to the average, we found no significant differences in evoked release amplitude and *t*_1/2_ of serotonin clearance (table in Figure 1).

### Evoked and Basal Serotonin in Male and Female Mice

**FIGURE 2:**
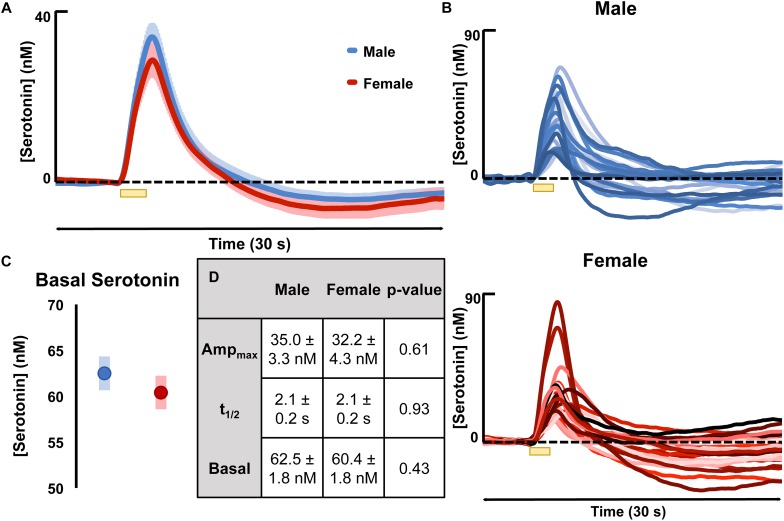
**(A)** The average evoked serotonin for male mice (blue, *n* = 23) and female mice (red, *n* = 23) with SEM shown in a lighter shade around each. Serotonin was evoked *via* a short stimulation (yellow bar). **(B)** The average basal serotonin concentration found in male (*n* = 17, shown in blue) and female (red, *n* = 18) animals is shown, along with the SEM of each. **(C)** The table details male and female average maximum evoked [serotonin] amplitude, the average t_1/2_ of serotonin clearance and the average basal [serotonin]. **(D)** The raw data for each individual mouse used to generate the average evoked serotonin signal are shown in varying shades of blue (male) and red (female). Significance was defined as *p* < 0.05.

Evoked serotonin was measured in male and female mice (regardless estrous cycle stage). Results are shown in Figure 2 where Figure 2A shows the average serotonin concentration ([serotonin]) vs. time traces in male and female mice and Figure 2B shows the raw data of each individual making up the averages shown in Figure 2A. The results of FSCAV experiments in male and female mice are seen in Figure 2C. The values for the maximum amplitude of serotonin release, the *t*_1/2_ of serotonin clearance, and the average ambient [serotonin] are in the table in Figure 2C (middle). These results demonstrate no significant difference in the evoked release amplitude (35.0 ± 3.3 nM in males and 32.2 ± 4.3 nM in females, *p* = 0.61, two-tailed student’s *t*-test), *t*_1/2_ of serotonin clearance (2.1 ± 0.2 s in males and 2.1 ± 0.2 s in females and *p* = 0.93, two-tailed student’s *t*-test), or average ambient serotonin levels [62.5 ± 1.8 nM in males and 60.4 ± 1.8 nM in females (*p* = 0.43, two-tailed student’s *t*-test)]. To determine if these results are specific to the hippocampus, we explored an additional brain region, the mPFC (Supplementary Figure S3). No statistical difference was found in this region between the male and female mice.

### Mathematically Modeling Male and Female Serotonin Signals

**FIGURE 3:**
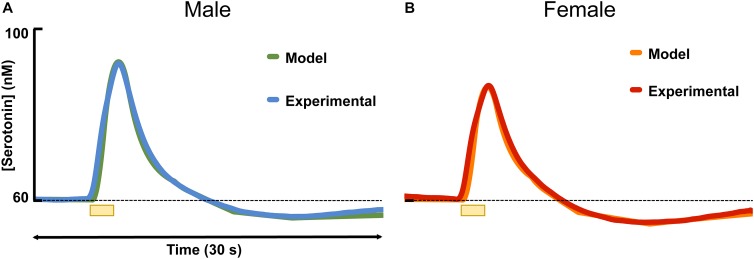
The average experimental serotonin signal from male **(A)** and female **(B)** mice is shown plotted with the modeled serotonin signal. An average basal value of 60 nM has been incorporated into the male and female responses.

Figure 3 shows the results of fitting the averaged male and female serotonin responses with a model that we previously developed that captures experimental data in the context of the synaptic mechanisms that control extracellular serotonin. In these animals that we reported evoked release, ambient serotonin data were not available. Thus, to give basis to the model, an average concentration from a subset of mice in this work (60 nM) was added, arbitrarily, to each signal. The amplitude of the signal in male mice was higher than in the female mice, which was modeled *via* a larger input term to the terminal (*r* = 0.54 in males, *r* = 0.43 in females). Additionally, there was a larger dip below baseline after stimulation in female mice, which was modeled *via* a larger value for the autoreceptor term in the model. We described this autoreceptor phenomenon at length previously (Wood et al., 2014).

### Serotonin Response to ESCIT

**FIGURE 4:**
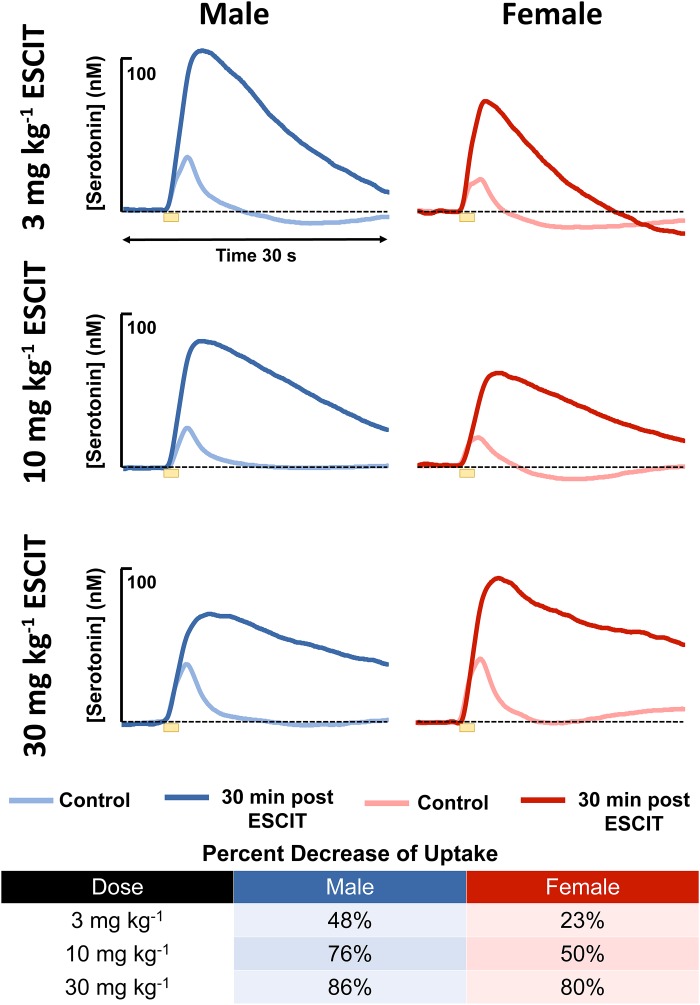
The male (blue) and female (red) evoked serotonin signals are shown (*n* = 4 for each). A lighter color is shown to indicate control release and a darker shade is used to denote response 30 min following i.p. ESCIT administration (3, 10, and 30 mg kg^-1^). The table lists percent decrease of reuptake in males and females at the three doses.

An acute ESCIT dose, was administered *via* i.p. injection, to male and female mice, separately at 3, 10, and 30 mg kg^-1^ and the evoked and basal serotonin responses were monitored in separate cohorts of mice per dose. The evoked responses, seen in Figure 4, are shown for each dose along with the percent change in reuptake. At every dose, the female mice had a lower percent change in reuptake compared to the males.

**FIGURE 5:**
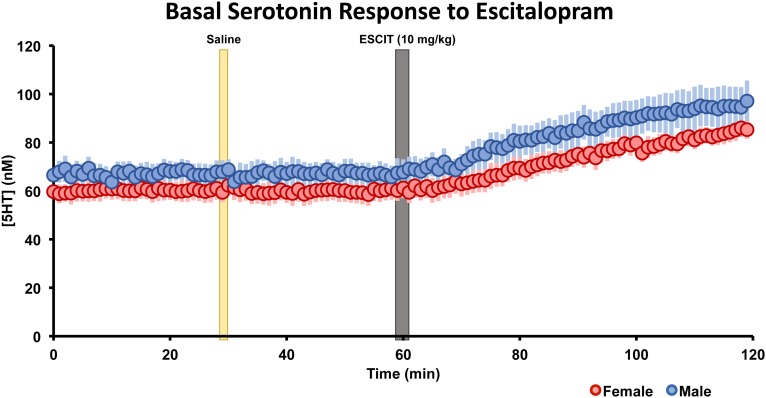
The male (blue, *n* = 5) and female (red, *n* = 5) basal serotonin levels were collected for 30 min. A saline injection (marked by a yellow bar) preceded another 30 min of basal serotonin data was collected. At the gray bar, escitalopram (ESCIT, 10 mg kg^-1^) was administered and 60 min of basal serotonin data was collection. SEM is shown as a lighter bar behind.

The basal, steady-state serotonin concentration response to ESCIT is shown in Figure 5 in male and female mice. Control files were collected for 30 min, after saline injection, files were taken for 30 min, finally ESCIT was administered and files were taken for an additional hour. Serotonin was not altered by saline, while roughly 10 min after ESCIT, the signal rose ∼43–46%. This represented a significant change in serotonin in both sexes (*p* < 0.01, two-tailed student’s *t*-test) but no significant difference between the two sexes (*p* = 0.96, two-tailed student’s *t*-test). Likewise, the basal serotonin concentrations before and after ESCIT were not significantly different between males and females (*p* = 0.244 and *p* = 0.220, respectively, two-tailed student’s *t*-test).

## Discussion

### Control Serotonin Chemistry Is Not Significantly Different Between the Sexes in the Hippocampus

The possible inherent biological underpinnings of depression are not well understood and hypotheses that have been brought forth over the years have not had unanimous recognition. It is well established that alterations in serotonin chemistry likely underlie the behavioral phenotypes of depression (Asberg et al., 1976a,b; Owens and Nemeroff, 1994; Stockmeier, 2003). We are in a good position to investigate the chemistry of the serotonin system from a fundamental perspective. Our techniques, FSCV and FSCAV, allow us to probe the essence of synaptic release and reuptake of serotonin *via* the many parallel systems that regulate this transmitters’ extracellular concentration.

There is a belief that neurochemistry in female rodents is intrinsically more variable due to the estrous cycle (Beery and Zucker, 2011). Accordingly, the majority of studies on serotonin’s role in depression and antidepressant effects (including our own) have so far only been in male mice (Borsini and Meli, 1988; Stahl, 1998; Wood and Hashemi, 2013; Abdalla et al., 2017). Here, we perform the first FSCV serotonin measurements in female mice and investigate whether the different stages of the estrous cycle alter the characteristics of serotonin neurochemistry. The estrous cycle in rodents is approximately 4–5 days long and is divided into four stages: proestrus, estrus, metestrus, and diestrus. These stages can be determined through looking at the difference in cell types in a vaginal smear under a standard light microscope and correlating those differences to the stage of estrous cycle (Caligioni, 2009; Byers et al., 2012) (Supplementary Figure S2). The phase of the estrous cycle for each female mouse was recorded and the responses of the mice in each respective cycle were averaged. Figure 1 shows that none of the averaged responses between the cycles differ statistically from the mean of 10 females blinded for stage of cycle. Estrogen is thought to modulate serotonin (Biegon et al., 1980; Rubinow et al., 1998), although it is unclear to which extent this phenomenon extends during the different stages of the cycle (Gundlah et al., 1998; Maswood et al., 1999; Bethea et al., 2002) Overall, our data do not have enough significance to support this notion in the hippocampus. Additional data from male and female mice in the mPFC (Supplementary Figure S3) also show no significant differences between the sexes. An important caveat here is that, while the hippocampus and mPFC are brain regions heavily implicated in depression and antidepressant actions (Goldapple et al., 2004; Kodama et al., 2004; Drevets et al., 2008), our study has not included other brain regions thought to be important in the pathology depression, which may show differences. An additional point of note is the age range of animals used here since there is evidence that depression rates vary with age (Anderson et al., 1987; McGee et al., 1992; Hankin et al., 1998; Piccinelli and Wilkinson, 2000). We utilized a broad age range (6–12 weeks) to maximize potential observable differences. In Supplementary Figure S1, we found no statistical differences between young adult (6–8 week old) and adult (9–12 week old) mice.

Scientists have found a variety of sex differences in rodents related to serotonin including: metabolism (Curzon and Bridges, 1970; Rosecrans, 1970; Kennett et al., 1986), synthesis (Nishizawa et al., 1997), receptor binding (Fischette et al., 1983; Arango et al., 1995; Parsey et al., 2002; Jovanovic et al., 2008), transporter functions (McQueen et al., 1997; Jovanovic et al., 2008), extracellular levels (Gundlah et al., 1998), and many other processes (Biegon et al., 1980; Rubinow et al., 1998; Dominguez et al., 2003). However, parallel studies have found little to no differences in other aspects of the serotonin system (Rosecrans, 1970; Kunimura et al., 2015). This incongruity makes it very difficult to ascertain whether the serotonin system plays a fundamental role in the disparity of depression between male and females.

In Figure 2A, we see that evoked release and reuptake of serotonin is remarkably reproducible in male vs. female mice. A point of note is the high precision of these experimental data. These data (Figure 2D) are essentially raw data (two analysis steps include smoothing the data and conversion of current to concentration). These data are highly reproducible, in contrast to other chemical data that often needs to be normalized and shown as a % change from baseline (Kaminska et al., 2018; Wojcieszak et al., 2018). Also, compared to dopamine voltammetry studies, where significant heterogeneity manifests as “hotspots” of dopamine activity (May and Wightman, 1989; Wightman et al., 2007), these serotonin data are much more uniform.

In Figure 2B, in the interest of scientific transparency, we purposefully show all of our raw data that were used to generate the average curves illustrated in the bottom panel. The SEM of the maximum amplitude of the average female curve is 4.3 nM. This small error is in accordance with our long-standing hypothesis that the serotonin system is a profoundly regulated one (Hashemi et al., 2009; Wood et al., 2014). Standard statistics showed no difference in the amplitude or *t*_1/2_ and of evoked release nor in basal [serotonin] (Figure 2). These statistical findings emphasize both the high reproducibility of our data and the tightly controlled *in vivo* serotonin system. To summarize these data: there are no statistical differences between the stages of the cycle and the mean in females. Critically, the averaged responses between male and female mice are not different in the CA2 region of the hippocampus.

### Modeling Serotonin Signals Reveals Potential Regulation Differences

We previously developed a model that deconstructs the chemical signal as a function of the many synaptic processes that control extracellular serotonin. Thus, modeling the experimental data can provide specific information about the components of system (Wood et al., 2014). As a starting point, we fit the averaged male and averaged female experimental data; the model hypothesized two differences between the male and female evoked response. First, the evoked response in males necessitated a larger model input term to the terminal. Second, a stronger autoreceptor term was necessary to model the female data. This finding is insightful but requires further verification since the model was based on the average, and not individual data. To further verify these notions, we hypothesized potential biochemical processes that underlie the findings.

#### Larger Model Input Term to the Terminal in Males

To model the experimental data, a larger input term was required in male mice to fit the larger amplitude of evoked serotonin release. We hypothesize three biochemical reasons for this larger input. First, a higher amplitude response could be the result of more axons. However, in most investigations, there have not been significant differences found in the number of serotonin axons between male and female mice (Jitsuki et al., 2009; Kunimura et al., 2015; Rajkowska et al., 2017). Second, we postulate that axons in male mice have a lower stimulation threshold than in female mice. The literature has come to no consensus on this front (Yang et al., 2015; Strupp-Levitsky et al., 2016). Third, we put forth that more vesicular serotonin is released in response to stimulation in male mice. This could be a result of a variety of changes within the synapse ranging from vesicle number, Ca^2+^ dependent vesicular release, amount of serotonin released with each vesicle fusion, or catabolism of released serotonin; again these possibilities remain inconclusive based on current literature (Mermelstein et al., 1996; Nishizawa et al., 1997; Rehavi et al., 1998).

#### Larger Model Autoreceptor Term in Females

Previously, we showed that the dip below baseline after stimulation was a fall in serotonin levels (Wood et al., 2014). We modeled and pharmacologically verified that this decrease in ambient serotonin level was due to prolonged autoreceptor control. In these data, the model captured a larger dip below baseline in females by necessitating a larger autoreceptor term. We postulate that this is a function of a higher density of, or higher functionality of autoreceptors in females. A significant amount of current research suggests sex may affect quantity and function of autoreceptors (Jones and Lucki, 2005; Goswami et al., 2010; Goel et al., 2014). Jones and Lucki (2005), in particular, determined that 5HT1B autoreceptor knockout mice exhibited a sex-dependent increase in baseline hippocampal serotonin present only in females. Goswami et al. (2010) found increased levels of 5HT1D autoreceptor mRNA in serotonin neurons in the dorsal raphe of females with major depressive disorder compared to control females, a trend that did not persist in male subjects.

The literature does not enable verification of the model’s two hypotheses, yet it is important to verify whether these fundamental differences in serotonin chemistry exist in the context of depression and/or antidepressant efficacy in males vs. females. As a pharmacological means to verify these hypotheses, we study acute methiothepin (non-selective serotonin receptor antagonist, with high affinity for the autoreceptors) and ESCIT administration.

### Escitalopram Induces Differences in Serotonin Uptake but Not Basal Concentrations Between Sexes

With the increasing global rate of depression, the issue of antidepressant efficacy is brought to the forefront. SSRIs have been shown to have different effects in male and female patients in clinical trials (Kornstein et al., 2000; Khan et al., 2005). In rodents, SSRIs have differential effects by sex. For example, male and female rodents respond differently in the forced swim test (FST) (Cryan et al., 2002; Drossopoulou et al., 2004; Petit-Demouliere et al., 2005; Bogdanova et al., 2013). The FST is a behavioral test; among other uses, it has been used to screen for antidepressant efficacy after acute i.p. SSRI injections in naive animals (Borsini and Meli, 1988; Willner, 1990).

In the context of antidepressant effects in male vs. female mice, we sought to test the two hypotheses brought forth by the model by adhering to the traditional SSRI screening procedure (acute SSRI i.p. injection). The first hypothesis was that there is a higher input to the serotonin terminals in male mice resulting in higher amplitude of evoked release. For the hypothesis to hold, this amplitude difference is expected to persist after SSRI. We used three different doses of ESCIT and presented averaged control evoked responses (*n* = 4 animals) and the averaged responses 30 min after SSRI administration. ESCIT administration slowed the reuptake of serotonin, as predicted by the mechanism of action of this agent. Notably, following ESCIT, there was a systematic increase in the signal amplitude; however, there was no consistency in amplitude change between the doses in the two sexes. Specifically, at 30 mg kg^-1^, the female ESCIT response amplitude is higher than that of the males. This finding nullifies the model’s first hypothesis, since the higher amplitude in males did not persist across all doses.

**FIGURE 6:**
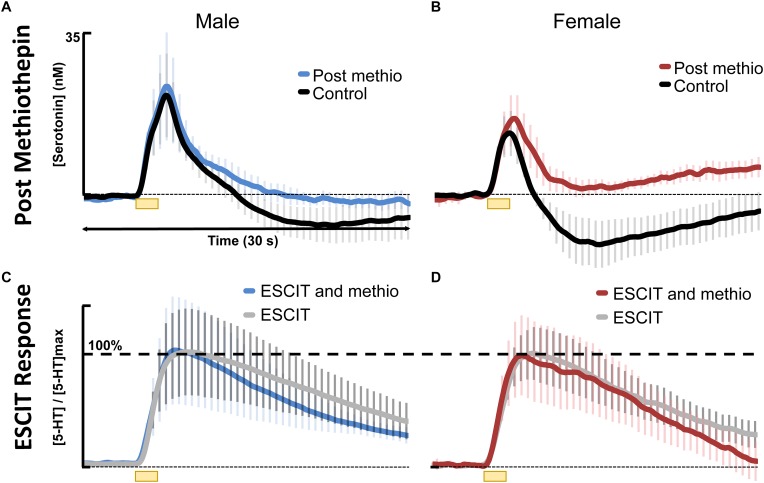
Control stimulated 5HT is shown in black for **(A)** males (*n* = 4) and **(B)** females (*n* = 4) and 30 min following 0.1 mg kg^-1^ methiothepin (methio) are shown for **(A)** males (*n* = 4) in blue and **(B)** females (*n* = 4) in red. Post methiothepin pretreatment ESCIT (10 mg kg^-1^) response is shown for **(C)** males in blue and **(D)** females in red. These are normalized to ESCIT responses seen in Figure 4, shown in gray for **(C)** males and **(D)** females.

In addressing the second hypothesis brought forth by our model, that there is stronger autoreceptor regulation in females, we found that acute ESCIT was less effective at decreasing the rate of serotonin reuptake at all doses in female mice. We propose that this phenomenon could be due to increased functionality of the serotonin autoreceptors, a notion supported in the literature (Jones and Lucki, 2005; Goswami et al., 2010; Goel et al., 2014). Increased autoreceptor activity could counteract SERT-mediated effects of the SSRI, especially since 5HT1-B autoreceptors are G-protein coupled to the SERTs and have previously been found to mediate serotonin reuptake (Montanez et al., 2014). A potential rationale for increased autoreceptor control in female mice is the modulation of serotonin by estrogen in the brain (Biegon et al., 1980; Lu et al., 1999; Bethea et al., 2002; Abraham et al., 2003; Sheng et al., 2004; Cornil et al., 2006; Barth et al., 2015; Kunimura et al., 2015), a complex relationship previously examined in in-depth reviews (Rubinow et al., 1998; Borrow and Cameron, 2014). Estrogen is thought to modulate serotonin in both male and female mice on both a slower, ambient level as well as rapid, transient effects which can alter intracellular signaling (Abraham et al., 2003; Cornil et al., 2006). The rapid changes in estrogen are more commonly associated with female models (Hansel and Convey, 1983). To protect against these “spikes” in estrogen, we propose the stronger activity of autoreceptors in female mice serve as a control mechanism. High levels of extracellular serotonin are neurotoxic (Boyer and Shannon, 2005). In accordance with this, other researchers have found increased density of 5HT1 autoreceptors and a higher rate of serotonin turnover in female vs. male mice, mediated by estrogen (Biegon et al., 1980; Carlsson et al., 1985). To test this notion, we pretreated male and female mice with methiothepine (0.1 mg kg^-1^) before ESCIT administration (Figure 6A,B). We were limited to using this small methiothepin dose because when administered with SSRI, receptor antagonists induce the fatal serotonin syndrome pathology. Statistically, we found that the “dip” below baseline, that we previously attributed to serotonin autoreceptors (Wood et al., 2014), is diminished in both sexes, but to a larger extent in female mice (191 ± 13 data points in the controls to 64.8 ± 45 files after methiothepin treatment, *p* = 0.048). This could point toward the fact that the female serotonin signal is under greater autoreceptor control since the same dose creates a larger effect (less of a correction is needed to prevent the “dip” in males). While these data are good evidence for stronger autoreceptor regulation in females, we find no effects of methiothepin pretreatment on the *t*_1/2_ of the ESCIT response (Figure 6C,D where responses are normalized from animals with and without methiothepin treatment). We thus reject the notion that autoreceptors are responsible for the lesser effect of ESCIT on the reuptake curve of serotonin in female mice. Other hypotheses that may account for this sex specific response to acute SSRI include metabolic effects (Curzon and Bridges, 1970; Rosecrans, 1970; Kennett et al., 1986), SERT trafficking (Malison et al., 1998; Joensuu et al., 2007), or promiscuous reuptake by other monoamine transporters (Fox et al., 2008; Daws, 2009). These effects will be the focus of future studies.

It is, however, important to note that the large electrical stimulations necessary to collect FSCV files cause aphysiological serotonin release. The stimulation allows us to test the nuances of the regulation of the serotonin system; however, it is unlikely that endogenous serotonin levels would spike to the levels we observe in our evoked experiments. Thus, we measured the ambient hippocampal serotonin response to ESCIT in male and female mice using FSCAV. We selected the intermediary dose of 10 mg kg^-1^, administered acutely. Interestingly, following administration of ESCIT, basal serotonin increased in both male and female mice with no detectable differences between the sexes (Figure 4).

Taken together, these data suggest that sex-mediated differences in serotonin reuptake following ESCIT in the context of stronger autoreceptor control in females is present only in response to the large electrical stimulation used with FSCV. This aphysiological stimulation allows us to delve into the fine biochemical differences that may exist within the synapse of male and female mice. These differences are in place to protect against potential variation in modulation between the sexes but are only activated under unusual circumstances. Therefore, on a physiological level, ESCIT appears to have consistent effects across the sex lines in mice.

## Conclusion

No distinction was found between the different stages of the estrous cycle, in the hippocampus, in female mice. Furthermore, the average male and female control serotonin signals and ambient levels were not significantly different from each other. Modeling the average male and female serotonin evoked signals revealed a larger autoreceptor effect in female mice, which was confirmed *via* methiothepin administration. It is important to note that these autoreceptor differences may only be visible under aphysiological conditions (such as electrical stimulation or disease). Nonetheless, we showed that autoreceptor effects were not responsible for our finding that acute SSRI are lesser able to slow reuptake in female mice. Therefore, while there is evidence that under physiological conditions, there are no differences in hippocampal serotonin; the data presented here emphasize considering sex as an important biological factor when evaluating disease states and personalized treatment options.

## Ethics Statement

This study was carried out in accordance with the recommendations of Institutional Animal Care and Use Committee (IACUC) at the University of South Carolina, which operates with accreditation from the Association for Assessment and Accreditation of Laboratory Animal Care (AAALAC). The protocol was approved by IACUC.

## Author Contributions

RS, MH, AB, and SB contributed to the collection of the data presented in this paper. MH, PH, AW, RS, MR, JB, and FN participated in writing the manuscript. MH, AW, and PH each worked on data processing and analysis. AW and MH created the figures and completed the necessary statistics for the data. MR, JB, and FN carried out the mathematical modeling of the data.

## Conflict of Interest Statement

The authors declare that the research was conducted in the absence of any commercial or financial relationships that could be construed as a potential conflict of interest.

## Supplementary Material

FIGURE S1 The raw data for evoked serotonin response in young adult mice (aged 6–8 weeks, *n* = (11 females, 3 males) = 14 mice total) and **(B)** adult mice (aged 9–12 weeks, *n* = (3 females, 5 males) = 8 mice total) are shown in purple and green respectively. **(C)** A table with the average maximum amplitude and *t*_1/2_ are shown, neither of which are statistically between the two age groups.

FIGURE S2 A representative cell sample pictomicrograph is shown for each of the estrous cycles: **(A)** estrus, **(B)** metestrus, **(C)** diestrus, and **(D)** proestrus. Estrus contains anucleated cornified cells, metestrus contains cornified, nucleated epithelial cells, and leukocytes, diestrus contains leukocytes and proestrus contains nucleated epithelial cells. Methods and analysis were completed as described in Caligioni, 2009.

FIGURE S3 Female (red) serotonin responses (*n* = 5) in the medial prefrontal cortex (mPFC) are compared to previously reported male (blue) serotonin response (*n* = 20). Error bars for every tenth file are shown in similar corresponding colors. The wide error bar range results from single and double peak signals being averaged together as well as the low n size in the case of the females. Nonetheless, the error bars suggest that there is no difference in male and female serotonin signals in the mPFC.
