# Corrigendum: *In vivo* Hippocampal Serotonin Dynamics in Male and Female Mice: Determining Effects of Acute Escitalopram Using Fast Scan Cyclic Voltammetry

**DOI:** 10.3389/fnins.2019.00726

**Published:** 2019-07-09

**Authors:** Rachel A. Saylor, Melinda Hersey, Alyssa West, Anna Marie Buchanan, Shane N. Berger, H. Frederik Nijhout, Michael C. Reed, Janet Best, Parastoo Hashemi

**Affiliations:** ^1^Department of Chemistry and Biochemistry, University of South Carolina, Columbia, SC, United States; ^2^Department of Pharmacology, Physiology, and Neuroscience, University of South Carolina School of Medicine, Columbia, SC, United States; ^3^Department of Biology, Duke University, Durham, NC, United States; ^4^Department of Mathematics, Duke University, Durham, NC, United States; ^5^Department of Mathematics, The Ohio State University, Columbus, OH, United States

**Keywords:** serotonin, hippocampus, SSRI, FSCV, FSCAV, sex, depression

In the original article, there was a mistake in the legend for Figure 5 as published. The legend for Figure 6 was accidently used instead. The correct legend appears below.

**Figure 5 |** The male (blue, *n* = 5) and female (red, *n* = 5) basal serotonin levels were collected for 30 min. A saline injection (marked by a yellow bar) preceded another 30 min of basal serotonin data was collected. At the gray bar, escitalopram (ESCIT, 10 mg kg^−1^) was administered and 60 min of basal serotonin data was collection. SEM is shown as a lighter bar behind.

The authors apologize for this error and state that this does not change the scientific conclusions of the article in any way. The original article has been updated.

