# Supplementary figures and images for: In vivo Hippocampal Serotonin Dynamics in Male and Female Mice: Determining Effects of Acute Escitalopram Using Fast Scan Cyclic Voltammetry
Source: Front Neurosci. 2019 Apr 23;13:362. doi: 10.3389/fnins.2019.00362 (PMC6499219; doi:10.3389/fnins.2019.00362)

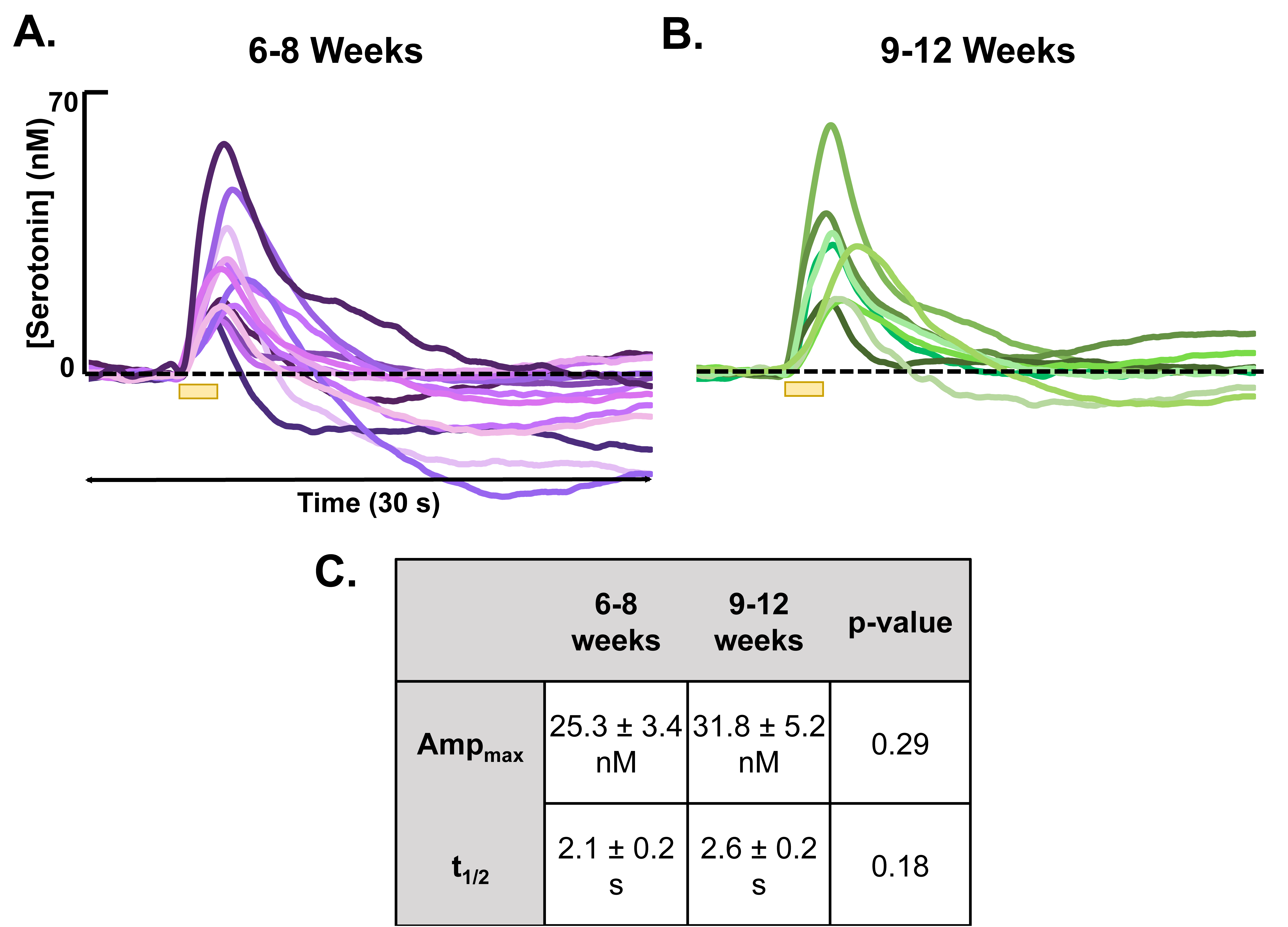

Supplement: FIGURE S1 — The raw data for evoked serotonin response in young adult mice (aged 6–8 weeks, n = (11 females, 3 males) = 14 mice total) and (B) adult mice (aged 9–12 weeks, n = (3 females, 5 males) = 8 mice total) are shown in purple and green respectively. (C) A table with the average maximum amplitude and t1/2 are shown, neither of which are statistically between the two age groups. [file Image_1.TIF]

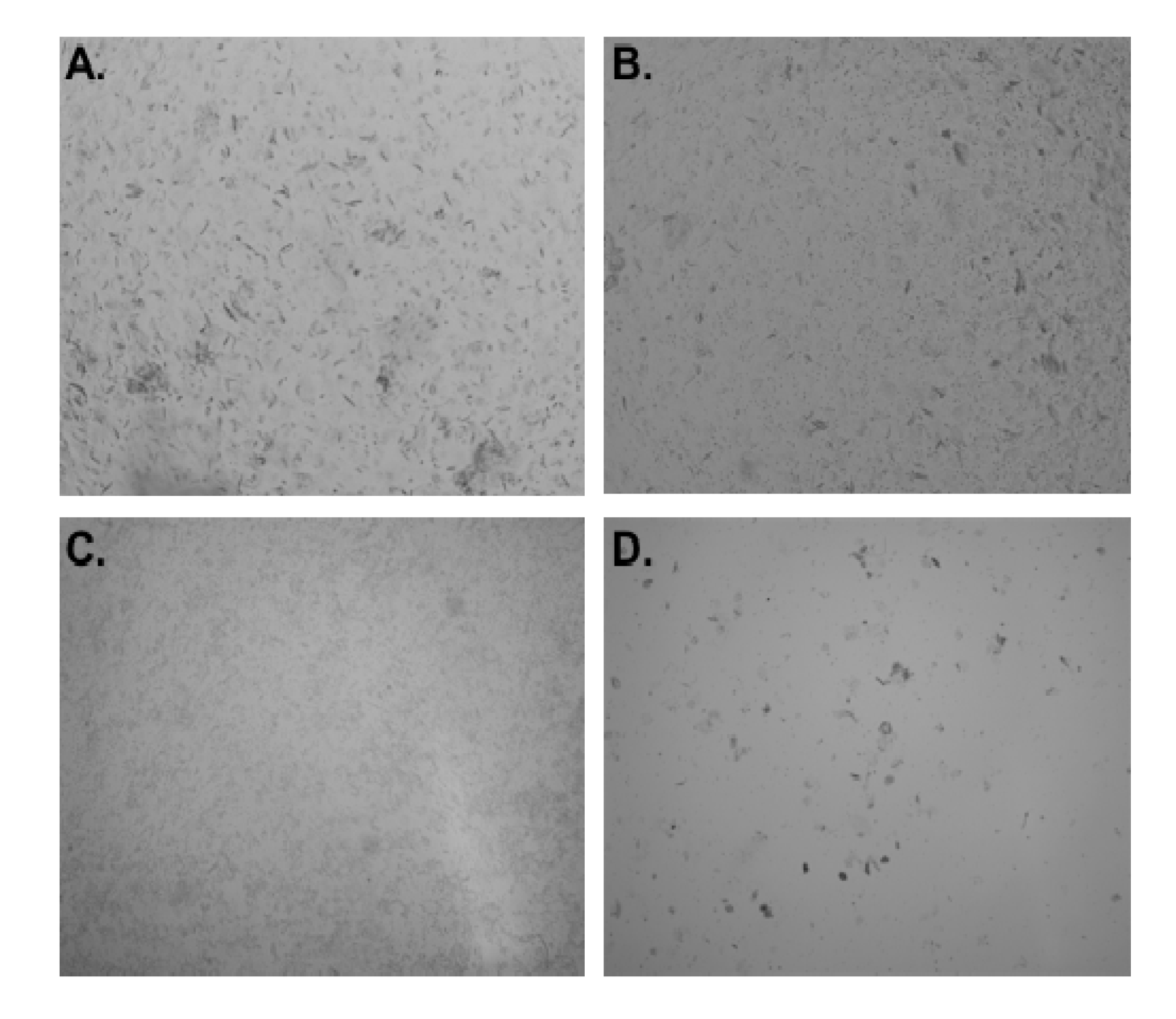

Supplement: FIGURE S2 — A representative cell sample pictomicrograph is shown for each of the estrous cycles: (A) estrus, (B) metestrus, (C) diestrus, and (D) proestrus. Estrus contains anucleated cornified cells, metestrus contains cornified, nucleated epithelial cells, and leukocytes, diestrus contains leukocytes and proestrus contains nucleated epithelial cells. Methods and analysis were completed as described in Caligioni, 2009. [file Image_2.TIF]

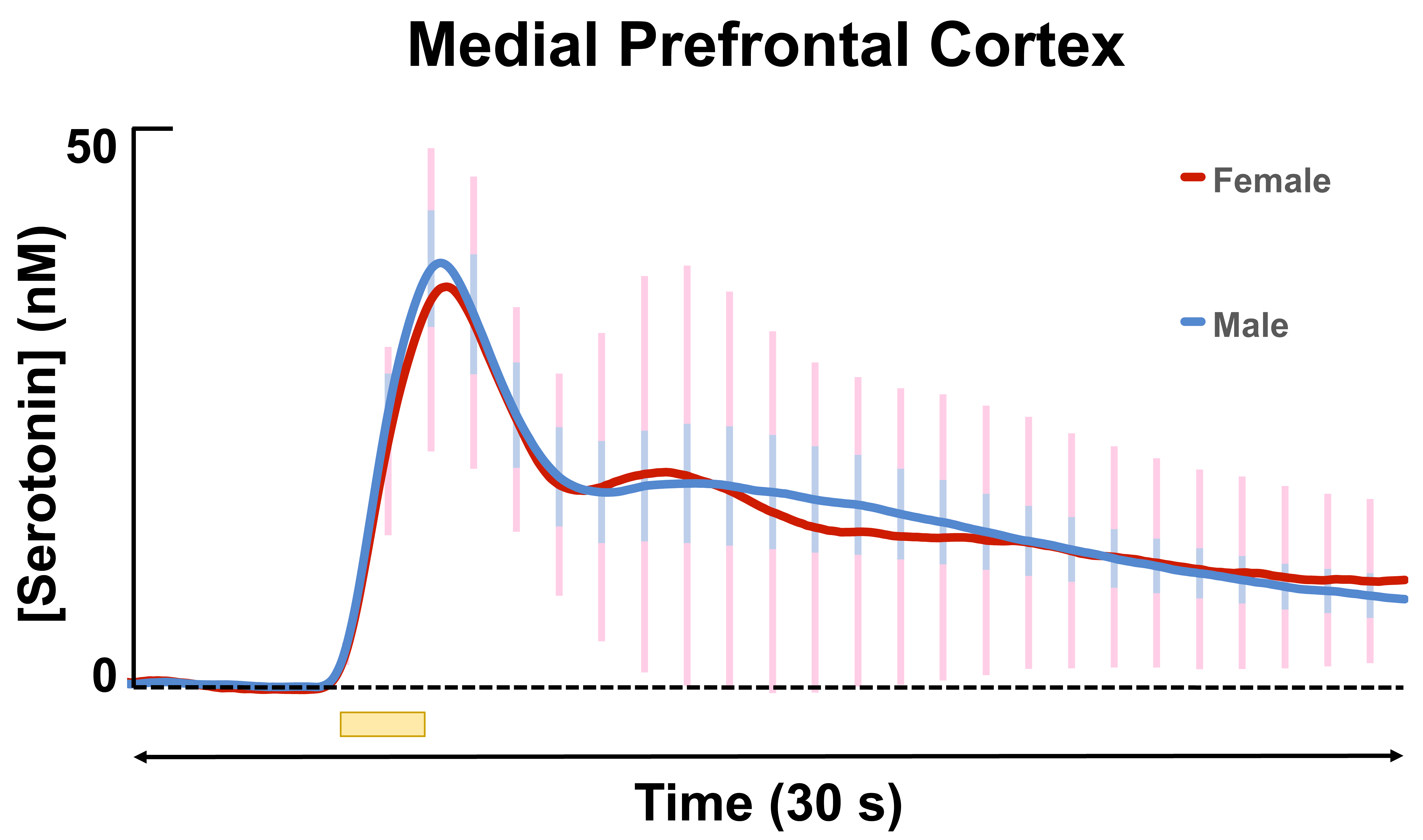

Supplement: FIGURE S3 — Female (red) serotonin responses (n = 5) in the medial prefrontal cortex (mPFC) are compared to previously reported male (blue) serotonin response (n = 20). Error bars for every tenth file are shown in similar corresponding colors. The wide error bar range results from single and double peak signals being averaged together as well as the low n size in the case of the females. Nonetheless, the error bars suggest that there is no difference in male and female serotonin signals in the mPFC. [file Image_3.TIF]
